# Immune Thrombocytopenic Purpura – Different Presentations in Two COVID-19 Patients

**DOI:** 10.7759/cureus.11202

**Published:** 2020-10-27

**Authors:** Ana Pedroso, Luciana Frade, Sara Trevas, Maria João Correia, Ana Luísa Esteves

**Affiliations:** 1 Internal Medicine Department, Hospital São Francisco Xavier, Lisboa, PRT; 2 Internal Medicine Department, Hospital Egas Moniz, Lisboa, PRT

**Keywords:** covid-19, glucocorticoids, immune thrombocytopenic purpura, sars-cov-2

## Abstract

Immune thrombocytopenic purpura (ITP) is a rare acquired autoimmune disease, resulting from platelet destruction and impaired platelet production. It has been described as associated with either genetic or environmental risk factors, such as viral infections, and in a few cases has been reported to be associated with severe acute respiratory syndrome coronavirus 2 (SARS-CoV-2).

Although steroid treatment is the most widely used first-line treatment of ITP, in the early days of coronavirus disease 2019 (COVID-19) it was controversial, but it has since become approved in treatment for COVID-19.

The authors report two different cases of COVID-19-associated ITP, with special emphasis on the timing of presentation, severity, and treatment decisions.

Remarkably, one of the patients who suffered severe thrombocytopenia was safely treated with corticosteroids in the late phase of COVID-19 infection.

## Introduction

ITP is a rare acquired autoimmune disease characterized by a platelet count under 100x109/L, resulting from platelet destruction and impaired platelet production.

It has been described to be associated with either genetic or environmental risk factors, including viral infections. It was previously reported to be associated with a different coronavirus strain [1]. Recently a number of cases of ITP have been reported associated with SARS-CoV-2 [2-9].

Despite being the most widely-used first-line treatment for ITP, steroid treatment was theoretically believed to carry an increased risk of infection in the COVID-19 context, so its use was depreciated over non-immunosuppressive treatments. Nonetheless, dexamethasone has been shown to lower mortality in patients with severe COVID-19 infection during the Randomised Evaluation of COVID-19 Therapy (RECOVERY) trial [10]. 

Recent guidelines ﻿of the British Society for Haematology (BSH) suggested that corticosteroids may be the best option for new or relapsed ITP in COVID‐19 patients and dosages and duration of the treatment should be the minimum necessary [11].

The authors report two patients with ITP associated with COVID-19, with different presentation and treatment.

## Case presentation

Clinical case one

A 67-year-old Caucasian woman presented to the emergency department with a two-week history of traumatic hip pain without pain control on analgesic therapy. She had no other symptoms, namely fever or respiratory complaints. At examination, she only had external right leg rotation. 

She had history of hypertension, cerebrovascular disease with previous ischemic stroke and a known exposure to a COVID-19 patient.

The radiography showed a subcapital right femur fracture, Garden IV. Laboratory blood tests were within the reference ranges and the nasopharyngeal swab polymerase chain reaction (PCR) test was positive for SARS-CoV-2. She was admitted to the COVID-19 ward for surveillance and orthopedic surgery.

On the fifth day of hospitalization she developed a cough which lasted for three days.

The Orthopaedic Surgery team decided to wait for negative SARS-CoV-2 testing considering she had no urgent surgery need. She was taking her chronic medication and on low molecular weight heparin (LMWH) for deep venous thrombosis prophylaxis.

On the 21st day, she started the pre-operative assessment and her blood tests showed isolated thrombocytopenia (platelet count 2.000 x109/L) (Table 1). She had skin blood suffusions on puncture sites. These findings prompted LMWH discontinuation and surgery was postponed.

**Table 1:** Patient characteristics and laboratory tests of the two patients with COVID-19-associated ITP (on the day of the platelet count nadir) ITP - Immune Thrombocytopenic Purpura; PT - prothrombin time; sec - seconds; APTT - activated partial thromboplastin time; PF4 - Platelet factor 4; ANA - Anti-nuclear antibody; HIV - Human Immunodeficiency Viruses; CMV - Cytomegalovirus; n.d. - not done

	Case 1	Case 2	Reference range
Age (years)	75	41	
Sex	Female	Female	
Day of symptoms	20	6	
Day of hospitalization	24	3	
Hemoglobin level (g/dL)	10,8	7,7	12.0 – 15.0
Platelet count (x 10^9^/L)	2.000	38.000	150 – 400
Leucocyte count (x 10^9^/L)	4,700	2,800	4.0 – 10
Lymphocyte count (x 10^9^/L)	1,87	0,8	0.5 – 5
PT (sec)	11.3	10.4	<14.0
APTT (sec)	29.0	22.8	23.0 – 38.0
D-dimer (ng/mL)	2.167	30.227	0 – 500
Direct coombs	n.d.	Negative	
Haptoglobin (mg/dL)	186	121	30-200
Plasmodium	n.d.	Negative	
Anti-heparin autoantibodies PF4	Negative	Negative	
Antiplatelets autoantibodies	Positive	Positive	
Lupic anticoagulant	Negative	Negative	
Anti-cardiolipin antibodies	Negative	Negative	
Anti-beta-2-glycoprotein I	Negative	Negative	
ANA	Negative	Negative	
HIV serology	Negative	Negative	
Hepatitis B	Negative	Negative	
Hepatitis C	Negative	Negative	
Parvo B19 virus	n.d.	IgG Positive, IgM Negative	
CMV virus	n.d.	IgG Positive, IgM Negative	

Prothrombin and activated partial thromboplastin times were normal and levels of thyroid peroxidase antibodies, antiplatelet factor 4, and antinuclear antibodies were not detected. Antiplatelet antibodies were positive.

She received initially a platelet transfusion, without any improvement.

Considering she was in the late phase of COVID-19, asymptomatic and without complications, prednisolone 1 mg per kilogram per day was started. Platelet counts reached normal levels within five days. The corticosteroid weaning and discontinuation was possible, maintaining normal platelet counts.

On the 41st day, she underwent bipolar right hip hemiarthroplasty, without complications. She started physical rehabilitation and was discharged home on the 48th day, with normal platelet counts.

Clinical case two

A 41-year-old African woman, with poorly controlled type 1 diabetes and stage 4 chronic kidney disease, presented at the emergency department with fever, myalgia, odynophagia, bilateral lumbar pain, dysuria, and vomiting. Physical examination was unremarkable. 

Laboratory blood tests showed pancytopenia and worsening renal function. PCR SARS-CoV-2 test was positive. She was admitted to the COVID-19 ward for surveillance, symptom control, and treatment.

Despite renal function improvement with hydration, thrombocytopenia progressively got worse (nadir of 38.000x109/L on the sixth day of COVID-19 symptoms) (Table 1). She had minor self-limited haemorrhagic complications on the puncture sites.

She had been under LMWH treatment, which was stopped due to the thrombocytopenia worsening.

The prothrombin and activated partial thromboplastin times were normal. A peripheral blood smear showed no schistocytes. Antiplatelet factor 4 and antinuclear antibodies were not detected. Antiplatelet antibodies were positive.

She was maintained hospitalized for surveillance and spontaneous total platelet recovery occurred on the 13th day of symptoms, without any specific treatment.

## Discussion

In the first patient, it was decided to initiate corticosteroids, considering she was in a late stage of COVID-19 infection, had severe thrombocytopenia, and needed a surgical procedure. This treatment allowed the patient full recovery without any immediate complication, enabling surgery.

The second patient did not receive corticosteroids and progressively recovered without any bleeding complication.

Although both patients were on LMWH, Antiplatelet factor 4 (Heparin antibodies) were not detected, which suggests other aetiology. Other causes of thrombocytopenia were also excluded. 

The other previously reported cases had also heterogeneous presentations (different phases of COVID-19 and severity) and several treatments and results [2-9]. Twelve out of 14 were treated with Immunoglobulin, five additionally with corticosteroids and two received platelet transfusions. Two of them died and 12 improved and were discharged. None of the patients treated with corticosteroids died [2-9].

Based on the RECOVERY trial [10], further recommendations were suggested supporting the use of steroids in COVID-19 patients [12]. However, they are not proven to be effective in patients who do not require supplemental oxygen. In these cases, its use is not recommended, unless a patient has another clinical indication for corticosteroid therapy. In the first patient there was indication supporting the use of steroids, with good results and no complications.

## Conclusions

These two cases emphasize that we should be aware of rare, unknown, and unexpected complications potentially related to COVID-19. The first one also supports the safety of corticosteroids in COVID-19 patients ITP treatment. However, there is no evidence supporting its use for COVID-19 patients who do not need supplemental oxygen and more studies are required.
